# Blended learning of radiology improves medical students’ performance, satisfaction, and engagement

**DOI:** 10.1186/s13244-020-00865-8

**Published:** 2020-04-28

**Authors:** Adrien Vavasseur, Fabrice Muscari, Olivier Meyrignac, Matthieu Nodot, Fabrice Dedouit, Paul Revel-Mouroz, Louis Dercle, Laura Rozenblum, Lucy Wang, Charlotte Maulat, Hervé Rousseau, Philippe Otal, Laurent Dercle, Fatima-Zohra Mokrane

**Affiliations:** 1 Service de Radiologie, CHU Toulouse-Rangueil, 1 avenue du Professeur Jean Poulhès, TSA 50032, 31059 Toulouse, Cedex 9 France; 2 Service de Chirurgie digestive, CHU Toulouse-Rangueil, 1 avenue du Professeur Jean Poulhès, TSA 50032, 31059 Toulouse, Cedex 9 France; 3 Ingénieur en pédagogie, service d’appui pédagogique, Université Toulouse III-Paul Sabatier, Route de Narbonne, 31300 Toulouse, France; 4 Service de médecine légale, CHU Toulouse-Rangueil, 1 avenue du Professeur Jean Poulhès, TSA 50032, 31059 Toulouse, Cedex 9, France; 5 Faculté de pharmacie de Châtenay-Malabry, 5 Rue Jean-Baptiste Clément, 92290 Châtenay-Malabry, France; 6 Sorbonne Université, Service de Médecine Nucléaire, AP-HP, Hôpital La Pitié-Salpêtrière, 75013 Paris, France; 7 Columbia University, 116th St & Broadway, New York, NY 10027 USA; 8 New York Presbyterian Hospital, Columbia University, New York City, NY USA; 9 Gustave Roussy Institute, UMR1015, Université Paris-Saclay, F-94805 Villejuif, France

**Keywords:** Education, Radiology, Medical students, Blended learning

## Abstract

**Purpose:**

To evaluate the impact of blended learning using a combination of educational resources (flipped classroom and short videos) on medical students’ (MSs) for radiology learning.

**Material and methods:**

A cohort of 353 MSs from 2015 to 2018 was prospectively evaluated. MSs were assigned to four groups (high, high-intermediate, low-intermediate, and low achievers) based on their results to a 20-MCQs performance evaluation referred to as the pretest. MSs had then free access to a self-paced course totalizing 61 videos based on abdominal imaging over a period of 3 months. Performance was evaluated using the change between posttest (the same 20 MCQs as pretest) and pretest results. Satisfaction was measured using a satisfaction survey with directed and spontaneous feedbacks. Engagement was graded according to audience retention and attendance on a web content management system.

**Results:**

Performance change between pre and posttest was significantly different between the four categories (ANOVA, *P* = 10^−9^): low pretest achievers demonstrated the highest improvement (mean ± SD, + 11.3 ± 22.8 points) while high pretest achievers showed a decrease in their posttest score (mean ± SD, − 3.6 ± 19 points). Directed feedback collected from 73.3% of participants showed a 99% of overall satisfaction. Spontaneous feedback showed that the concept of “pleasure in learning” was the most cited advantage, followed by “flexibility*.*” Engagement increased over years and the number of views increased of 2.47-fold in 2 years.

**Conclusion:**

Learning formats including new pedagogical concepts as blended learning, and current technologies allow improvement in medical student’s performance, satisfaction, and engagement.

## Key points

- Low achievers take the best advantages from blended learning using video-based lectures.
- The use of a SPOC-based learning in a blended learning format was associated to a high face-to-face optional course attendance (86.1%) highlighting thus the high students engagement.
- While directed feedback informs on students’ overall satisfaction; spontaneous feedback provides a better understanding of the mechanisms that influence students’ learning: Pleasure in learning is a major point in students’ adherence to a new learning format.

## Introduction

Past decades were highly impacted by rapid technological advancements and the introduction of information and communication technologies (ICT). This change led to permanent economic, social, and environmental changes, making nowadays society information-driven and highly connected [1]. This radical shift in societal behavior also reflects in students’ and teachers’ behaviors and must be considered by current educational guidelines. New educational methods must be dynamic and responsive, in order to follow this evolving environment [2].

E-learning formats from massive open online courses (MOOC) to short private online courses (SPOC) are widely used in educational sciences, including medical education courses [3, 4]. This paradigm shift in learning methods is a disruptive force because it challenges the tradition of lectures and shifts the educational experience in a learner-centered way. Health professions use SPOC technologies to improve the professional medical education of all medical students (MSs), ranging from undergraduate to postgraduates’ trainees [1, 5–8]. These design decisions are driven by economical, logistical, and other planning considerations. However, most of time, the decision is made based on the combined strength of different modalities for presenting course information [3]. Indeed, SPOCs’ widespread acceptability has led to revised policies in educational standards, whatever are their types, ranging from fully online elements, i.e., computer-based learning environment without classroom components, to primary face-to-face learning with minor online elements [8].

Blended learning (BL) refers to a new educational strategy that integrates traditional face-to-face academic sessions with synchronous or asynchronous e-learning, to support and improve meaningful interactions between students, teachers, and electronic resources [9]. Most commonly, BL takes the advantages of both traditional courses and SPOC. Flipped classroom (FC) is one of the most challenging parts of BL, where learners are introduced to new concepts via independent use of “pre-work” from SPOC [10]. This newly acquired knowledge is then applied and consolidated in an interactive classroom [11, 12]. This educational concept has the advantage of allowing students to engage the information of a discipline, at their own pace [13–15]. SPOC delivery platforms allow educators to create and deliver interactive courses in many forms as online resources, quizzes, virtual patients, or video-based lectures (VBL) [16–18]. Many undergraduate MSs are auditors who engage primarily with videos while skipping over assessment problems, online discussions, and other interactive course components [19]. They, as young adults from Generation Z, make an extensive use of video in their daily lives [20]. The use of level-adapted VBLs is a new and innovative concept that meets the expectations of both teachers and students [21].

The importance of developing a formal medical imaging program has been demonstrated worldwide [22]. Indeed, medical imaging teaching is often sporadic and taught irregularly during other medical modules rather than on its own [5]. Medical school educational programs, particularly radiology undergraduate training, are not standardized throughout Europe and USA [23–25]. The educational benefits of different types of radiology clerkships point at improvement in students’ knowledge, interpretation skills, and students’ satisfaction [26]. Additionally, there is a gap in knowledge of the exact benefits of these new educational tools compared to classical ones. Indeed, literature data lacks of reproducible and reliable tools to evaluate these techniques, and in the way to interpret results [1, 9, 27]. In this context, undergraduate medical imaging learning has been restructured in local university hospital using a combination of these new teaching concepts: blended learning based on a FC delivered in the form of VBLs and a face-to-face optional course. The purpose of this study is to decipher the impact of this teaching formula among three MS promotions. For this, three types of metrics were evaluated—performance, satisfaction, and engagement—using both quantitative metrics (pre/posttest results’ differences, students’ attendance, audience retention, and key moments of audience overview) and qualitative assessment criteria (directed and spontaneous feedback).

## Material and methods

### Study design

This single-site prospective study was conducted in a University Hospital Center between July 2015 and May 2018.

**Fig. 1:**
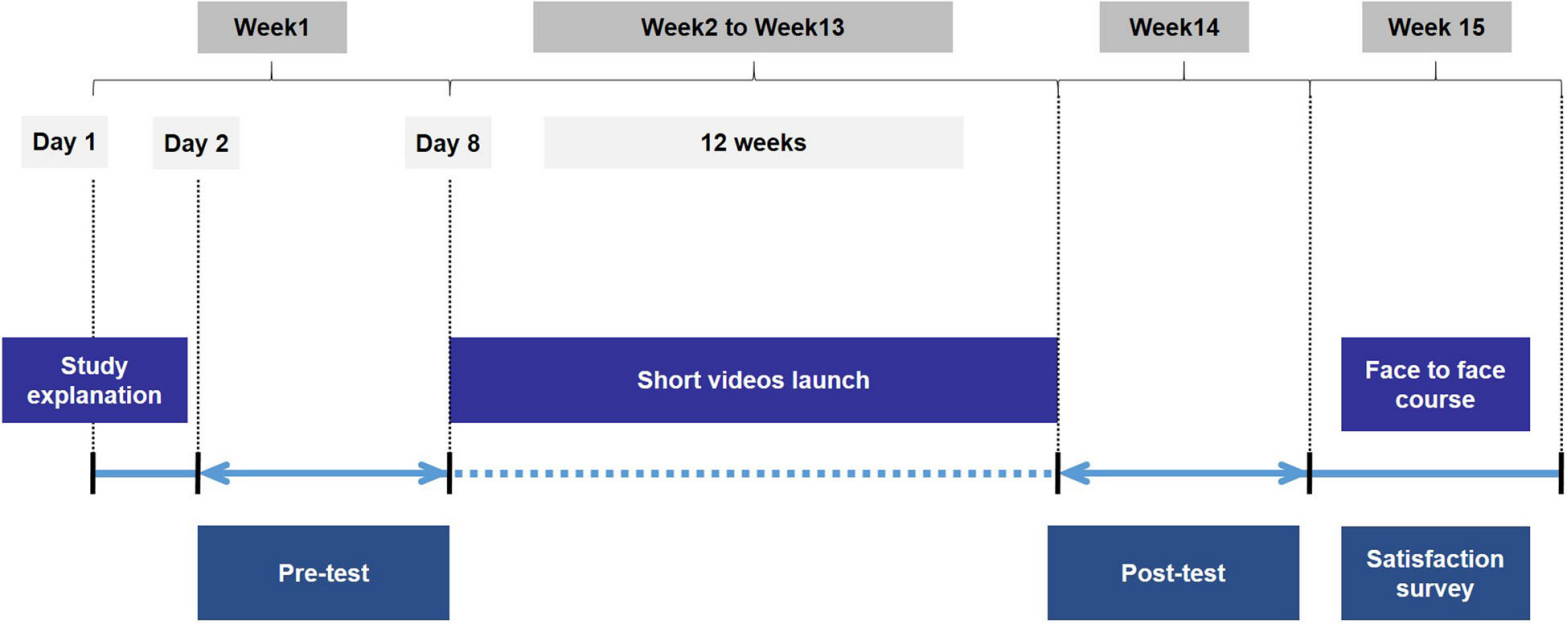
Study design and timeline

Figure 1 shows the study design. For three consecutive years, the study started the first day of abdominal rotation for 4th year undergraduate MSs (day 1). First, two teachers (AV and FZM) presented the study to students during a short talk. Then, an initial online assessment (pretest) was launched and was available for 1 week (week 1*)*. After pretest closing (week 2), VBLs were launched on a web content management system, and were available for 3 months. During week 14, a final online assessment (posttest) was launched.

All MSs who conducted the pre and posttests were included in the study. The final step of the study was a 2-hour optional face-to-face course during the last week (week 15), in which test answers were discussed with students. At the end of this course, a facultative satisfaction survey was distributed to students.

Institutional Review Board approval was waived since the research involves no risk; the research did not involve use of identifiable private information, and did not adversely affect the rights and welfare of the subjects. Pedagogical faculty committee approved the study design.

### Pedagogical material creation (VBLs)

Two experienced radiologists (AV and FZM) created short videos dedicated to this learning format. AV and FZM are experienced in both abdominal imaging (3 and 10 years of experience respectively) and in medical teaching (2 and 5 years of experience respectively). Several criteria were respected during VBL creation. First, all content was based on national medical imaging and gastroenterology MSs educational guidelines [28, 29]. Second, all included routine medical images originated from local PACS (picture archiving and communication system) and were strictly anonymized in compliance with French legislation. Finally, several *form specificities* were taken into consideration, according to international educational recommendations [30]: (i) video type used should be lectures with conceptual knowledge, produced using slide presentations with voice over; (ii) the requirement for video length is less than 6 mn, except if it contains medical imaging video loops (less than 15 mn); and (iii) videos must contain several pictures and imaging examples, video-motions with real imaging examples and continuous visual flow, along with extemporaneous speaking and extensive use of arrow pointer. All videos were reviewed by an expert radiologist, with 25 years of experience in abdominal imaging and 15 years of experience in medical teaching (PO).

### Content management system

Videos were launched using a free open source web content management system [31] designed to provide educators and learners with a system dedicated to creating personalized learning environments. For each promotion, a dedicated moodle session, linked to our university website, was created. Online personal registration was mandatory for each MS.

### Evaluation

Three educational parameters (performance, satisfaction, and engagement) and different types of metrics were used in this study in order to evaluate the obtained results quantitatively and qualitatively.

#### Performance evaluation: pre/posttest evaluation

The primary endpoint was to evaluate the impact of such a learning format on different types of students according to their performances, in order to know its impact on low achievers. For this, difference between pre and posttests results were calculated and compared in each student’s category. Pre and posttests consisted of the same 20 multiple-choice questions (MCQs), focused on abdominal imaging. Tests were launched using a dedicated medical examination website [32]. The pre and posttests were scored on a 100-point scale distributed identically among the 20 MCQs*.* The mean overall improvement between the pre and posttests was calculated. Then, the overall population was divided into quartiles according to pretest scores (high, high-intermediate, low-intermediate, and low achievers) to evaluate the effectiveness of VBL according to pretest ranking.

#### Satisfaction survey evaluation

As a secondary endpoint, students’ opinions on this learning format were collected. During the optional face-to-face course (week 15), a satisfaction survey composed of two parts was provided to all attendants.

The first part of the survey consisted of directed feedback. Answers to 13 questions were ranged using a 5-point Likert scale. The rating scale for the overall course scoring was 1 = poor/strongly disagree, 2 = below average/disagree, 3 = average/neither agree or disagree, 4 = above average/agree, and 5 = excellent/strongly agree.

Then, as a part of a qualitative assessment, students were asked to give a spontaneous feedback by writing 2 to 3 sentences in answer to the question: “What is your overall perception of this learning format, with emphasis on positive points and points of improvement?”. Answers were then collected as positive or negative concepts according to answers.

#### Engagement evaluation

The last secondary endpoint was to evaluate the engagement of students on the three consecutive promotions. Engagement indicators, extracted from Moodle platform statistics, were as follows: (i) student attendance with number of overall views, (ii) audience retention report for each video, and (iii) key moments of audience overview.

### Statistical analysis

Qualitative variables were reported using counts and frequencies (percentages). Quantitative variables, following a Gaussian distribution in our study, are described by their means and standard deviations. Scores are expressed in absolute value and range from 0 (minimum) to 100 points (maximum). Students were divided into four quartiles (referred as [low, low-intermediate, high-intermediate, high] achievers) based on the pretest score. The efficacy of our teaching strategy was evaluated by computing differences between pretest and posttest. To this end, we have subtracted the absolute value of the pretest score from the absolute value of the posttest score. Pretest and posttest were also compared using Bland-Altman test. Chi-square test was used to compare scores between pre and posttests. Correlations between the pretest and posttest were assessed using Spearman’s rho. ANOVA analysis was used to compare scores for each quartile. Student’s evaluation of engagement was scored using the Likert scale (ranging from 1 to 5). Scores 4 and 5 using Likert scale (agree and strongly agree to the statement) were considered as “very satisfied.” Statistical analyses were performed using the SPSS software (version 23.0, commercially available, IBM, Armonk, NY) and R software (version 3.6.0, open source).

## Results

### Participants

Four hundred thirty-three MSs, distributed through three consecutive promotions/years, were potentially eligible for this study. Among them, 353 (81.5%) MSs performed both pre and posttests, and were included in the study accordingly (Table 1).

**Table 1:** Overall selected medical students

	Promotion 1 (year 2015/2016)	Promotion 2 (year 2016/2017)	Promotion 3 (year 2017/2018)	Total
Overall (eligible students)	148	149	136	433 (100.0)
Students performing the pretest	146	140	132	418 (96.5)
Students performing the posttest	133	118	111	362 (83.6)
Students performing took both pre and posttests*	127	116	110	353 (81.5)*
Students attending the optional course	102	93	109	304 (86.1)**
Students answering the survey	68	88	103	259 (73.3)**

Data are expressed in number of citations. Data in parentheses are percentages.

*Students included in the study. **Percentages are expressed regarding only included students

### Video-based lectures

A total of 61 videos grouped in 17 major topics were recorded. Video lengths ranged from 1 min 17 s to 12 min 18 s (Table 2). The average length was 5 min 5 s and the total duration was 5 h 11 min 1 s.

**Table 2:** Length of each video topic and student’s attendance to videos during 3 months**

VBLs’ topics	Number of videos	Duration*	Overall	Promotion 1	Promotion 2	Promotion 3
Appendicitis	6	4 min 56 s, [3 min 1 s–8 min 34 s]	99	77	83	142
Diverticulitis	2	7 min 43 s, [3 min 7 s–12 min 18 s]	84	64	75	117
Peritonitis	2	7 min 58 s, [5 min 56 s–9 min]	83	67	75,5	110
Acute intestinal occlusion	5	5 min 37 s, [3 min 13 s–7 min 31 s]	76	65.2	69	95
Gastrointestinal bleeding	2	4 min 52 s, [3 min 51 s–5 min 52 s]	74	56	71	96.5
Colorectal tumors	3	3 min 35 s, [4 min 28 s–8 min 44 s]	71	65.33	71.33	78
Esophagus tumors	2	5 min 16 s, [5 min 12 s–5 min 20 s]	70	54	61	97
Inflammatory bowel disease	3	4 min 18 s, [1 min 56 s–6 min 15 s]	70	54	70.33	89
Acute abdominal pain	2	4 min 59 s, [3 min 3 s–6 min 54 s]	70	59	63.5	89
Acute and chronic pancreatitis	7	5 min 5 s, [1 min 33 s–8 min 36 s]	69	48	52.5	112
Gastro duodenal ulcer	1	6 min 55 s	68	57	60	89
Gallbladder disease	4	4 min 15 s, [2 min 18 s–5 min 29 s]	67	51	54.25	98
Gastric tumors	1	7 min 31 s	64	53	60	81
Hernias	1	7 min 12 s	64	48.33	62	84
Cirrhosis	6	5 min 30 s, [2 min 11 s–6 min 16 s]	56	48	53.4	68
Liver tumors	12	4 min 23 s, [1 min 17 s–6 min 49 s]	56	51.5	54.75	63
Pancreatic tumors	2	6 min 25 s, [6 min 15 s–6 min 34 s]	46	35	37.5	67

*Data are expressed in average [minimal duration–maximal duration].

**Average student’s attendance during 3 months per video for each topic (week 2 to week 15)

### Performance of pre and posttests

**Fig. 2:**
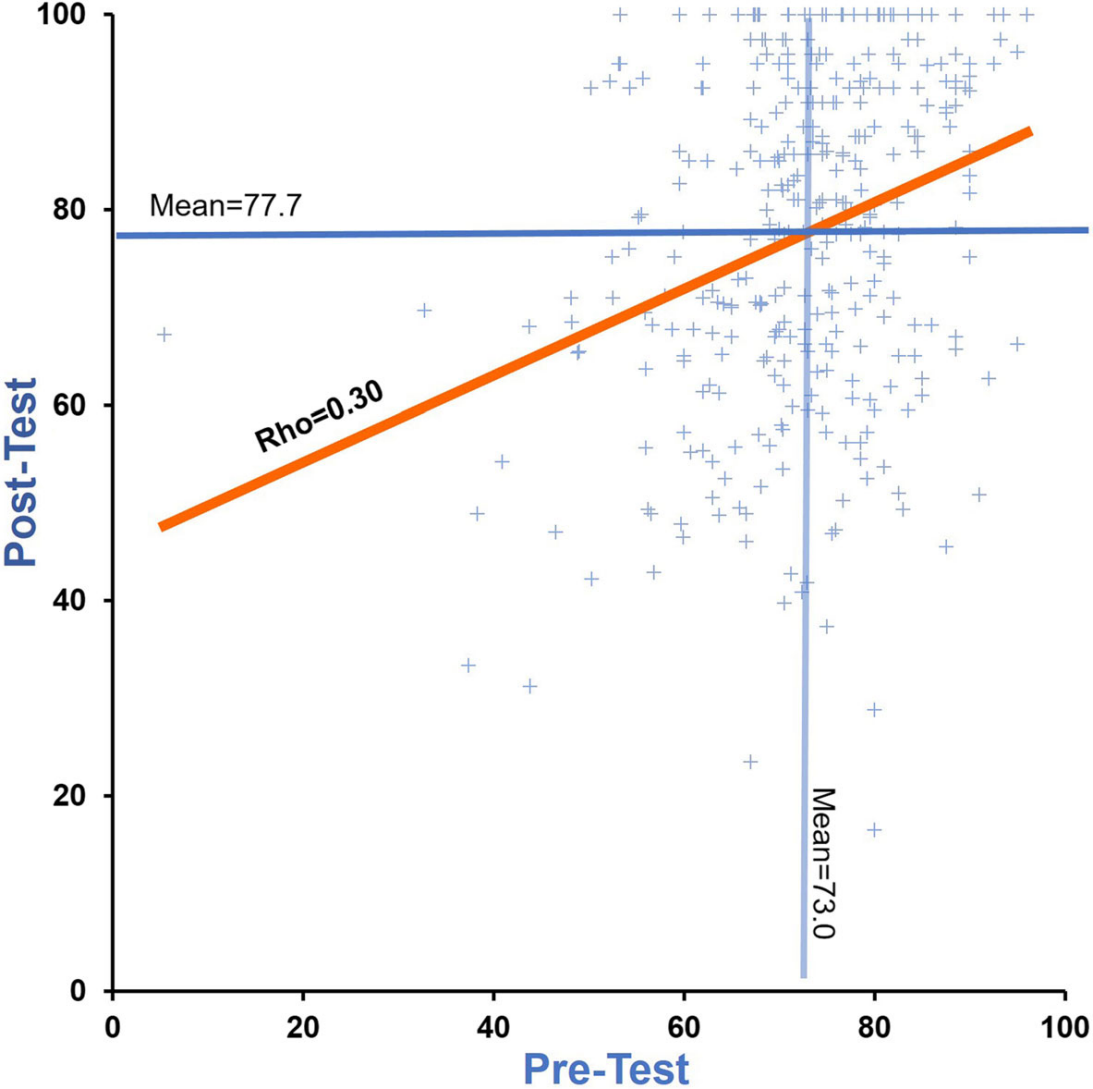
Evaluating performances: pretest vs. posttest comparison using Bland-Altman test. The average score was 73.0 for pretest and 77.7 for posttest. The absolute change was therefore an increase of 4.7 ± 17.5 points overall (*P* < 0.05). There is a correlation between the average value overall and the improvement between the pretest and posttest (Spearman’s rho = 0.39)

**Fig. 3:**
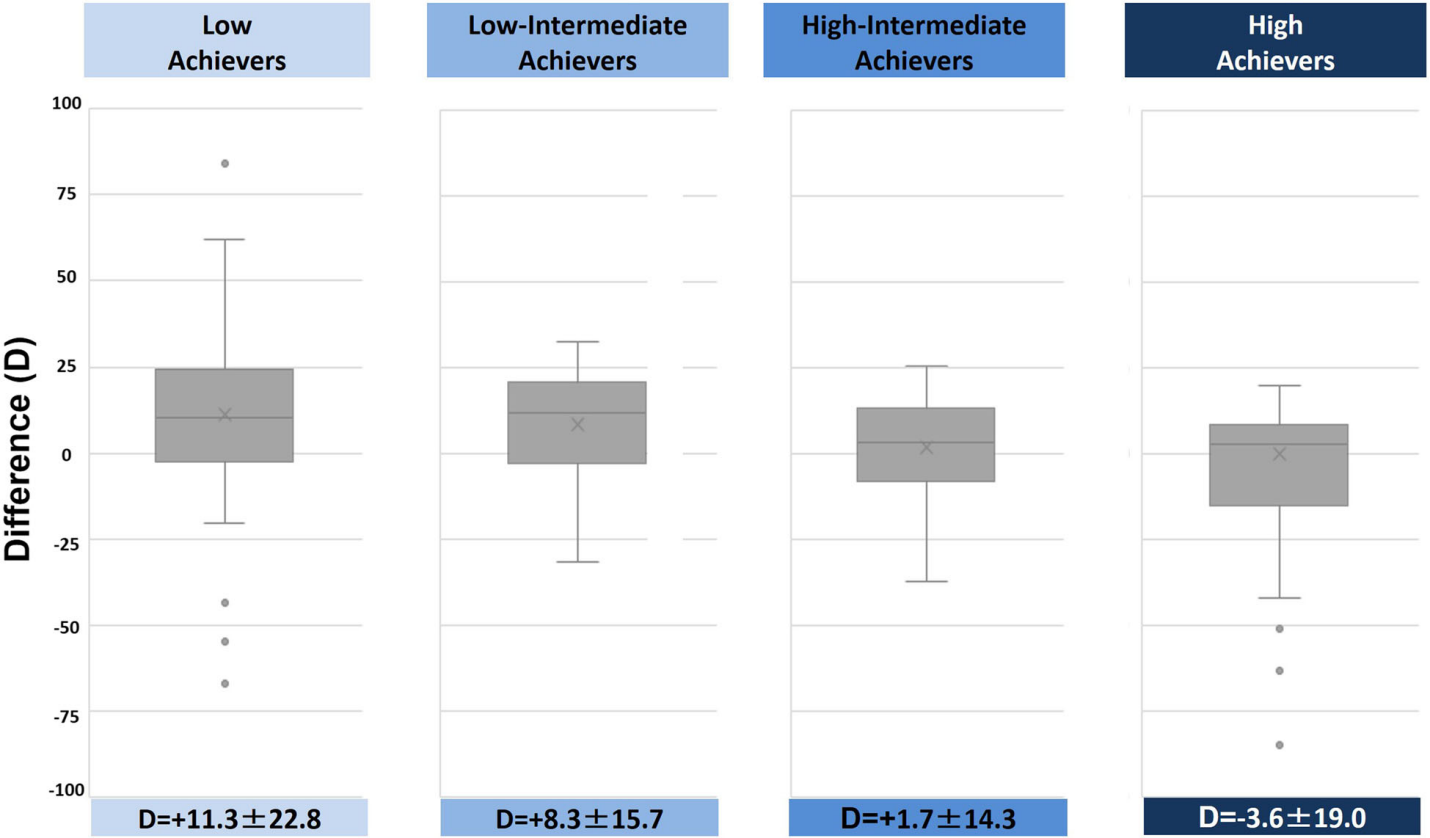
Comparison of pre and posttest results according to groups defined by pretest scores. Difference = (posttest score)−(pretest score). The highest improvement was observed in low achievers, 11.3 points ± 22.8 (*P* = 10^−9^). Difference (D) is expressed in mean ± standard deviation

The mean overall change between the pre and posttests was statistically significant (+ 4.7 points, *P* = 10^−9^). There was a moderate correlation between pre and posttests (Rho = 0.30, *P* < 0.05) (Table 3, Fig. 2)*.* The change in performance between the pretest and the posttest was significantly different between the four categories of achievers (ANOVA, *P* = 10^−9^): low pretest achievers demonstrated the highest improvement (mean: + 11.3 points, SD: ± 22.8 points) while high pretest achievers showed a decrease in their posttest score (mean: − 3.6 points, SD: ± 19 points) (Table 3, Fig. 3).

**Table 3:** Students’ results and distribution according pretest results quartiles

	*n*	Pretest*	Posttest*	Delta overall*
Overall	353 (100.0)	73.0 ± 12.1	77.7 ± 17.6	+4.7 ± 17.5
Quartiles**				
Quartile 1				
Low achievers	89 (25.2)	57.2 ± 12.7	65.6 ± 24.6	+ 11.3 ± 22.8
Quartile 2				
Low-intermediate achievers	88 (24.9)	70.7 ± 7.7	75.6 ± 22.3	+ 8.3 ± 15.7
Quartile 3				
High-intermediate achievers	90 (25.5)	76.8 ± 8.4	72.6 ± 25	1.66 ± 14.4
Quartile 4				
High achievers	86 (24.4)	85.7 ± 9.9	82.1 ± 21.3	− 3.6 ± 19
*P* values**		NA	*P* = 10^−7^	*P* = 10^−9^

Data are expressed in number of students (*n*), percentage, and mean ± standard deviation. *Scores are expressed in absolute value for a 100-point test.**ANOVA analysis for each quartile

### Satisfaction survey evaluation

Among the 304 students (86.1% of included students) that attended to the optional face-to-face course, 259 completed the optional satisfaction survey (73.3% of the included students, Table 1).

#### Directed feedback

Ninety-nine percent of MSs (*n* = 257) rated the overall learning experience as above average to excellent (Likert scores of 4 and 5). Ninety-five percent of them (*n* = 245) considered that they improved their knowledge using this method. Moreover, 99% (*n* = 256) recommended this learning format to students in other medical imaging fields (Table 4).

**Table 4:** Directed feedback collected from students during the face-to-face optional course

Satisfaction survey	Promotion 1*	Promotion 2*	Promotion 3*	Total*
**Format evaluation**				
Did this learning format encourage you to study?	66 (97%)	82 (93%)	93 (90%)	241 (93%)
Was the preliminary work reasonable?	51 (75%)	82 (93%)	89 (86%)	222 (86%)
Do you consider the platform suitable?	43 (63%)	81 (92%)	92 (89%)	225 (87%)
**VBL evaluation**				
Are the videos easily accessible?	63 (93%)	88 (100%)	102 (99%)	253 (98%)
Are the videos clear?	68 (100%)	83 (94%)	101 (98%)	254 (98%)
Are the videos adapted to your level of knowledge?	65 (96%)	84 (95%)	100 (97%)	249 (96%)
Are the videos of an adapted duration?	65 (96%)	77 (88%)	94 (91%)	236 (91%)
Did you like the format of the videos?	62 (92%)	86 (98%)	101 (98%)	249 (96%)
**Tests evaluation**				
Did you improve using this learning method?	64 (94%)	82 (93%)	99 (96%)	245 (95%)
Did you review reference books?	67 (99%)	81 (92%)	91 (88%)	239 (92%)
Did you have the opportunity to self-evaluate?	57 (84%)	77 (88%)	86 (83%)	220 (85%)
**Global satisfaction**				
Are you satisfied with this learning format?	68 (100%)	88 (100%)	101 (98%)	257 (99%)
**Perspective**				
Would you recommend this learning format to other medical imaging fields?	66 (97%)	87 (99%)	103 (100%)	256 (99%)

*Represented student evaluation corresponds to scores 4 and 5 using Likert scale (agree and strongly agree to the statement)

#### Spontaneous feedback

Over 1525 spontaneous feedback reports were provided (average, 6 feedback per MSs). The concept of pleasure in learning was cited 750 times (49.2% of overall feedback, average of 3 times per student), describing the learning format as engaging (102 citations) and motivating (130 citations). This was followed by the concept of flexibility (314 citations), whereas the concept of performance enhancement and time optimization was cited only 200 times (Table 5)*.*

**Table 5:** Spontaneous feedback collected from students during the face-to-face optional course

	Overall *n* = 259	Promotion 1 *n* = 68	Promotion 2 *n* = 88	Promotion 3 *n* = 103
**Concept of pleasure in learning**	**750**	**216**	**234**	**300**
Engaging	102 (38.6)	38 (55.8)	24 (27.3)	40 (38.8)
Adapted format	110 (42.4)	19 (27.9)	41 (46.6)	50 (48.5)
Motivating	130 (50.2)	35 (51.5)	43 (48.9)	52 (50.5)
Didactic	120 (46.3)	50 (73.5)	30 (34.1)	40 (38.9)
Clear	119 (45.9)	30 (44.1)	38 (43.2)	51 (49,5)
Correspond to their needs	169 (65.3)	44 (64.7)	58 (65.9)	67 (65.0)
**Concept of flexibility**	**314**	**81**	**95**	**138**
Enhances autonomy	218 (84.2)	60 (88.2)	68 (77.3)	90 (87.4)
High availability	96 (37.0)	21 (17.6)	27 (30.7)	48 (46.6)
**Points needing improvement**	**261**	**84**	**71**	**106**
Extend the content to other imaging learning	65 (24.9)	11 (16.2)	23 (26.1)	31 (30.0)
Technical adjustment (bad sound, inappropriate background color)	52 (20.0)	18 (26.5)	12 (16.9)	22 (21.4)
Lack of courses dedicated to normal abdominal imaging	75 (28.6)	6 (8.8)	27 (38.0)	42 (40.8)
Need for material support (PDF, downloaded videos)	69 (26.6)	49 (72.0)	9 (10.2)	11 (10.7)
**Concept of performance and time optimization**	**200**	**72**	**55**	**73**
Allows progression	128 (49.4)	49(72.0)	37 (42.0)	42 (40.8)
Time-saving	72 (27.8)	23(33.8)	18 (20.5)	31 (30.1)

Data expressed in number of citations. Data in parentheses are percentages for each promotion

Points needing improvement were cited 261 times, leaded by their request for more normal abdominal imaging (75 citations) and the possibility of access to material support (69 citations).

### Engagement evaluation

In order to evaluate the students engagement in the new pedagogical method tested in this study, three different aspects of engagement were analyzed.

#### Students’ attendance

**Fig. 4:**
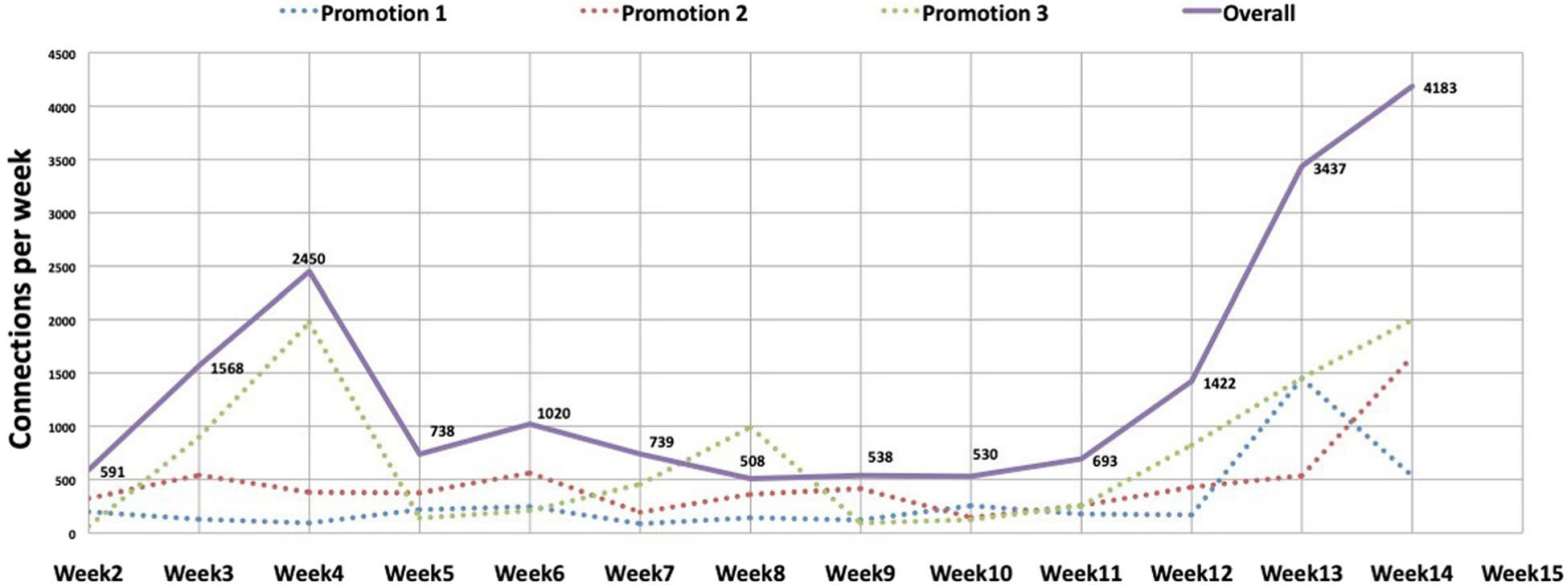
Number of connections during each week of VBL availability. Two peaks are observed. After pretest (week 4) and before posttest (week 14)

Each student viewed 55.2 videos during the study. During promotion 1, the 127 MSs have viewed 3838 times the videos. This number of views has increased during the following promotions (6182 views by 116 MSs during promotion 2, and 9482 views by 110 MSs during promotion 3), showing thus a 2.47-fold increase in the number of connections in 2 years (Fig. 4).

#### Audience retention

Over a 3-month period, the topic totalizing the most important views was “Appendicitis” (99 average views per video), followed by “Diverticulitis” (84 average views per video), and “Peritonitis” (83 average views per video). Topics focusing on chronic diseases such as cirrhosis, liver tumors, and pancreatic tumors were among the less consulted videos (Table 2).

#### Audience overview: key moments

Videos were most consulted at 2 key moments during the study: the beginning of the study (after VBLs launching and pretest) and before the posttest (Fig. 4).

## Discussion

Learning medical practice requires young MSs to develop practical skills while increasing their knowledge, in order to be prepared for their future profession [33]. During the second cycle of medical studies, students learn how to interact not only with patients and their diseases, but also with the entire medical care team [34]. Additionally, the medical knowledge required for their future exercise is broad, covering fundamental knowledge, practical knowledge, particular gestures, and human contact [35]. For the last decades, new pedagogical techniques were tested in all medical fields, with the clear purpose of increasing students’ knowledge and commitment [36, 37].

Comparison of pre and posttests results showed a significant overall improvement (+ 4.7 ± 17.5, *P* = *P* = 10^−9^). Moreover, result analysis according to quartiles revealed that the low-achieving group (the lowest quartile) was most impacted by this learning. Indeed, while the vast majority of MSs improved in their posttest performances, low achievers showed the most important improvement. SPOCs delivered in the form of VBLs have important advantages in medical learning: speed of installation, availability, and no need for physical presence [38]. However, a successful blended learning combining several educational concepts requires that MSs adhere to VBLs and watch videos prior to the class. Metrics from this type of formula can be difficult to collect and interpret. In this study, quantitative results comparing pre and posttests were explored in a new manner. Low achievers appropriated VBL and their results have approached those of high achievers allowing for a homogenization in students’ improvement. Social cognitive theory emphasizes self-efficacy as the primary driver of motivated action, and identifies cues that influence future self-efficacy and support self-regulated learning [39]. While high achievers are encouraged and highly praised, less academically inclined students are often marginalized [40]. Low achievers usually report more positively toward the use of video as a learning tool, perceived as more effective learning [12]. Regardless of their level, VBL allows every MS to progress. These benefits are more apparent in low achievers, as shown in this study. High achievers seem to be reinforced by their preset results, and probably focused on other learning tasks or other courses, adapting thus their own learning schedule.

The students were globally very satisfied regarding VBLs (99% of satisfaction), as well as regarding the final face-to-face course. The pedagogical formula used in this study achieved high student engagement and satisfaction. Indeed, 86.1% of included students attended to the optional face-to-face course, showing their interest in keeping a close contact with teachers. E-learning has a major impact on Generation Z students [41]. Its use in teaching radiology for MSs is widely documented, with global student and teacher satisfaction as well as results improvement [39, 40, 42, 43]. The objective is a clear trend toward a highly interactive and self-directed learning environment to support the concept of life-long independent learners [44]. However, the community of teachers has difficulties finding scientific evidence on how they should design their teaching [45, 46].

FC presents benefits of both e-learning and traditional teaching, allowing for a greater educational impact with less instructional time [42, 47]. Students can access learning materials at their own pace [48]. In medical imaging learning, this technique seems to be more effective than traditional learning, but requires greater rigor and commitment from students [27], and a high quality of online imaging content and quizzes [49]. In this study, the overall rating of the learning format by students was high (99% of level of satisfaction), despite the higher required commitment. This is explained by the fact that VBLs were perceived as “engaging” by students. Indeed, pleasure in learning is a key concept for e-learning. This concept represents 750 citations in the spontaneous feedback collected by the satisfaction survey in this study. Furthermore, motivation is a fundamental criterion in online education. The absence of interaction with teachers makes it difficult to learn alone remotely, especially for complex courses and requires the establishment of more and longer seminars [50]. In our study, students clearly expressed the increase in their motivation related to VBLs. Motivation due to impending deadlines is important, as seen with the peak in student engagement and video connection peak before the posttest. Indeed, induced motivation results in a more extensive use of VBL by MSs than self-determined motivation [51]. However, further studies with true video engagement quantification will help to better understand students’ expectations.

In this study, there is a clear value in new educational technologies with a blended learning format combining e-learning and a face-to-face classical course, in which students can be in direct interaction with their teachers. Indeed, students improved their results, adhered to the learning concept, and attended to the face-to-face course. In current literature data, there is a lack of extensive and quantitative studies in medical learning in highlighting the real impact of e-learning [52, 53]. Although there are strong pedagogical arguments that favor a blended learning approach, literature on the relative effectiveness of blended versus traditional learning approaches is mixed. Students’ attendance and adherence to BL seem to be higher when compared to a classical format; this type of teaching favors long-term memorization and knowledge assimilation [13–15, 54, 55], without impact on outcomes [56–58].

VBLs achieving the highest view counts are those concerning emergency topics (appendicitis and diverticulitis) that may be applicable to a broader range of medical learners. The ability to access the educational content immediately, regardless of location, appears to be a major concern for students. Radiology is becoming increasingly important in the diagnostic and therapeutic management of patients. Indeed, radiology education does not only concern future radiologists but also any future doctors. Medical practitioners need more and more imaging knowledge upon time and technologies [59]. The development of mobile-learning, by the implementation of videos on smartphone applications, makes it possible to overcome this concern while using an innovative learning tool that is appreciated by the students [60–62].

Educational videos are widely used in educational science as a dissemination tool for web-based training [63]. The effective use of videos as a teaching tool is enhanced when teachers consider how they should manage the cognitive load of videos, maximize student engagement with videos, and promote active learning from video [64]. The choice of using short videos in this work (average length of 5 min 5 s) was based on the fact that engagement is highly associated with video length [65]. The main advantage of the VBL format is time and place flexibility for potentially overwhelmed learners. Our choice of video production style was driven by our main purpose, which was to provide massive real-life imaging examples of both normal and pathological imaging findings in abdominal imaging [66]. Slide presentations with voice over production style showed high engagement in this study (96% of satisfaction).

However, this study has some limitations. First, there is an inclusion bias: 18% of MSs did not perform pre and posttests (80/433) and only 259 students over the 353 included (73.3%) answered the satisfaction survey. This is mainly due to the fact that these tests were not included in the final note that validates the abdominal rotation. In order to reduce the “missing” students’ rates, several reminders were introduced during the entire rotation, in order to encourage the students to perform the whole experience.

Further studies, in which true video engagement quantification is measured, and comparison to classical face-to-face courses will help to better understand students’ needs and behaviors regarding new technologies.

## Conclusion

The blended learning format used in this study, combining short video-based lectures, as flipped classroom material, with a face-to-face course and pre and posttests showed high results regarding students’ performance, satisfaction, and engagement. This learning method has allowed students to progress by improving their motivation and pleasure in learning. This study shows that several metrics can be used to measure students’ improvement, and furthermore, the reasons for the success of a specific learning format, in a conceptual manner.

## Data Availability

All data and materials can be available online.

## Abbreviations

BL: Blended learning
FC: Flipped classroom
MCQ: Multiple-choice questions
MS: Medical students
SPOC: Small Private Online Course
VBL: Video-based lectures
